# Influence of Gel Stage from Cellulose Dissolution in NaOH-Water System on the Performances of Cellulose Allomorphs-Based Hydrogels

**DOI:** 10.3390/gels8070410

**Published:** 2022-06-29

**Authors:** Diana Elena Ciolacu, Daniela Rusu, Raluca Nicoleta Darie-Niţă, Daniel Tîmpu, Florin Ciolacu

**Affiliations:** 1 Department of Natural Polymers, Bioactive and Biocompatible Materials, “Petru Poni” Institute of Macromolecular Chemistry, 700487 Iasi, Romania; 2 Department of Physics of Polymers and Polymeric Materials, “Petru Poni” Institute of Macromolecular Chemistry, 700487 Iasi, Romania; rusu.daniela@icmpp.ro; 3 Department of Physical Chemistry of Polymers, “Petru Poni” Institute of Macromolecular Chemistry, 700487 Iasi, Romania; darier@icmpp.ro (R.N.D.-N.); dtimpu@icmpp.ro (D.T.); 4 Department of Natural and Synthetic Polymers, “Gheorghe Asachi” Technical University of Iasi, 700050 Iasi, Romania

**Keywords:** cellulose allomorphs, gel stage, gel fracturing, cross-linking, hydrogels, rheology

## Abstract

Novel hydrogels were prepared starting from different cellulose allomorphs (cellulose I, II, and III), through a swelling stage in 8.5% NaOH aqueous solution, followed by freezing at low temperature (−30 °C), for 24 h. After thawing at room temperature, the obtained gels were chemical cross-linked with epichlorohydrin (ECH), at 85 °C. The swelling degrees of the hydrogels were investigated, and a complex dependence on the type of the cellulose allomorph was found. Moreover, the gel stage has been shown to play a key role in the design of hydrogels with different performances, following the series: H-CII > H-CI > H-CIII. The correlations between the allomorph type and the morphological characteristics of hydrogels were established by scanning electron microscopy (SEM). The hydrogel H-CII showed the biggest homogeneous pores, while H-CIII had the most compacted pores network, with small interconnected pores. The rheological studies were performed in similar shear regimes, and a close correlation between the strength of the gel structure and the size of the gel fragments was observed. In the case of hydrogels, it has been shown that H-CII is softer, with a lower resistance of the hydrogel (G′) above the oscillation frequencies tested, but it maintains its stable structure, while H-CIII has the highest modulus of storage and loss compared to H-CI and H-CII, having a stronger and more rigid structure. The X-ray diffraction (XRD) method showed that the crystalline organization of each type of allomorph possesses a distinctive diffraction pattern, and, in addition, the chemically cross-linking reaction has been proved by a strong decrease of the crystallinity. Attenuated total reflectance Fourier transform infrared (ATR-FTIR) spectroscopy provided clear evidence of the chemical cross-linking of cellulose allomorphs with ECH, by the alteration of the crystal structure of cellulose allomorphs and by the formation of new ether bands.

## 1. Introduction

Since their development in the late 1960s, hydrogels have distinguished themselves by a network-like three-dimensional (3D) structure, as well as by their unique properties, which gives them competitive advantages compared to the other materials, making them a promising material-platform for various applications [1,2,3]. The undeniable versatility of hydrogels in terms of their synthesis, composition, and physical and chemical properties has led to a considerable attention from both research and industry and has created wide applicative potential, from the agricultural and industrial to the biomedical field. A key feature of hydrogels is their ability to absorb large quantities of water, which makes them soft, pliable, and compatible with most of the living tissues and thus of certain interest for the biomedical field [4,5]. In addition, their viscoelastic nature reduces any possible damage to the surrounding tissue, after potential implantation [6]. Moreover, the mechanical properties are comparable to those of soft tissue, a feature which renders them particularly attractive to tissue engineers [7].

Using the natural polymers in the preparation of the hydrogels represents an attractive option over the most of the synthetic counterparts, due to the wide design possibilities of some specific compositions and properties, providing, besides the support function, key features such as biodegradability, biocompatibility, and low latent toxicity [8,9]. Moreover, hydrogels based on natural polymers can be employed either for the sustained and controlled (stimuli-responsive systems) delivery of active pharmaceutical principles (mainly drugs and proteins) to different cultures and tissues, or as scaffolds for tissue engineering [10,11,12,13,14].

In this regard, cellulose, the main constituent of plants fibers and the most abundant natural polymer, with benefits such as low cost, renewability, availability, biodegradability, and non-toxicity, represents one of the most frequently used polymers in the preparation of hydrogels [15]. Cellulose and its derivatives have proven to be versatile polymers, which provide a good platform for the building of 3D networks and various possibilities to design the physico-chemical features of hydrogels, in terms of mechanical behavior, swelling ability, and sensibility to external stimuli [7,9,16].

Cellulose displays a highly ordered crystalline structure, based on extensive intra- and intermolecular hydrogen bonds, and is able to crystallize into various allomorphic forms that differ in unit cell dimensions and chain polarity (cellulose I, II, III, and IV) [17,18]. The structural differences of cellulose allomorphs, in terms of crystalline and amorphous contents and the sizes and shapes of the crystallites corresponding to the allomorphic forms of cellulose, are considered to be important parameters in the realization of functional materials.

Cellulose I (CI), or native cellulose, represents the prevalent form in plants. This contains highly ordered (crystalline) structures, made of tightly packed linear chains, disposed of in a parallel arrangement and held together by inter- and intramolecular hydrogen bonds and also, disordered regions (amorphous) [18]. The natural form of cellulose was found to be a composite of two coexisting crystalline forms, such as cellulose Iα (a triclinic lattice with one chain per unit cell) and cellulose Iβ (a monoclinic lattice with two chains per unit cell) [19]. Cellulose II (CII) is obtained by regeneration from various media or by mercerization in an aqueous NaOH solution [20,21]. CII displays an anti-parallel arrangement of the chains and possesses a superior chemical reactivity, as compared to cellulose I. Moreover, the transition from cellulose I to cellulose II is not reversible, and this implies that cellulose II is a stable form as compared with the metastable cellulose I. Another crystalline form, known as cellulose III (CIII), can be derived from both cellulose I and II, using different treatments [22,23,24]. Thus, CIII can be prepared either by soaking cellulose samples in anhydrous liquid ammonia at −80 °C or in organic amine.

Each of these three allomorphic forms of cellulose, with the advantages and limitations of their structural architectures, which are reflected in their accessibility and reactivity, present a wide and interesting array of potential applications in different domains.

The variations found between the crystalline and amorphous regions, within the cellulose structure, are the result of both the origin and history of the applied treatments. Many reagents are able to penetrate only the amorphous part of the material, and most of the reactions will take place in this area or at the interface with the crystalline ones. Thus, to increase the chemical reactivity of cellulose it is important to make more accessible the crystalline regions of cellulose to reagents, by swelling and de-crystallization methods.

Due to the fact that important applications of cellulose involve its regeneration in several morphologies such as fibers, films, membranes, nanoparticles, or bulky objects (sponges and aerogels), the dissolution of cellulose is a crucial and challenging step [25,26]. Cellulose is difficult to dissolve in water or most organic solvents due to its characteristic structure with close packing of macromolecular chains and a strong intra- and interchain bonding network [18,27,28]. More recently, this common concept that cellulose–cellulose hydrogen-bonding is the main obstacle to dissolution and the driving force of aggregation has been questioned and, instead, it was suggested that hydrophobic interactions play a very important role in cellulose (in)solubility [27].

There are a limited number of solvents that can directly dissolve cellulose, and between these, the most important ones are the phosphoric acid-based solvents, LiCl-based solvents, N-methyl morpholine N-oxide/water, ionic liquids, and NaOH–water [29,30,31,32]. Nevertheless, most of these solvents are limited to a laboratory scale due to issues such as toxicity, environmental hazard, and limited solvency [33].

One of the most common and attractive solvents of cellulose is the NaOH–water-based system, which has gained attention due to its low cost and of the fact that is easily recyclable and environmentally friendly.

A number of publications have reported that NaOH-water system can be a good solvent for cellulose, if certain conditions are met. These conditions are: (i) the concentration of the alkaline solution, which must be in the range of 7–10% NaOH solution, and (ii) the temperature, which must be low enough, even below 0 °C. As a result of the accomplishment of these conditions, it was proved that the NaOH-water system is able to break the intra- and intermolecular hydrogen bonds [30,34,35,36,37].

The dissolution of cellulose in NaOH-water can be achieved to a certain extent depending on factors such as the molecular weight and the dimensions of the crystalline domains [30,33,38,39]. However, most of these alkaline water-based systems allow only for the dissolution of celluloses with a relatively low degree of polymerization, DP < 300.

Isogai and Atalla [39] investigated the possibility of dissolving various types of native cellulose (microcrystalline cellulose, linter cellulose, softwood unbleached, and bleached kraft pulps and softwood groundwood pulp) and of different chemically treated cellulose (mercerized and regenerated). They observed that dissolution is possible only if cellulose is suspended in a solution of 8.5% NaOH, at a low temperature of −20 °C. After the thawing of the frozen mass at room temperature, water is added over the obtained gel, in order to reach a solution containing 2% cellulose in 5% aqueous NaOH.

Considering the above-mentioned dissolution method [39] and the high applicative potential of the hydrogels, the authors intended to obtain hydrogels with controlled densities, porosities, and swelling degrees, starting from the allomorphic forms of cellulose with different crystalline structures.

Thus, the aim of this study is to evaluate the influence of the gel structure obtained from the cellulose-8.5% NaOH system, based on the Isogai and Atalla method, on the performance of their corresponding hydrogels, prepared by chemical crosslinking with epichlorohydrin (ECH).

The hydrogels were obtained starting from three allomorphic forms of cellulose (cellulose I, II, and III) and were investigated by means of swelling measurements, X-ray diffraction (XRD) analysis, scanning electron microscopy (SEM), attenuated total reflectance Fourier transform infrared (ATR-FTIR) spectroscopy, and oscillatory rheology, in order to establish their structure and morphology, as well as the swelling and rheological behaviors.

## 2. Results and Discussion

### 2.1. Swelling Behavior of Hydrogels

**Figure 1:**
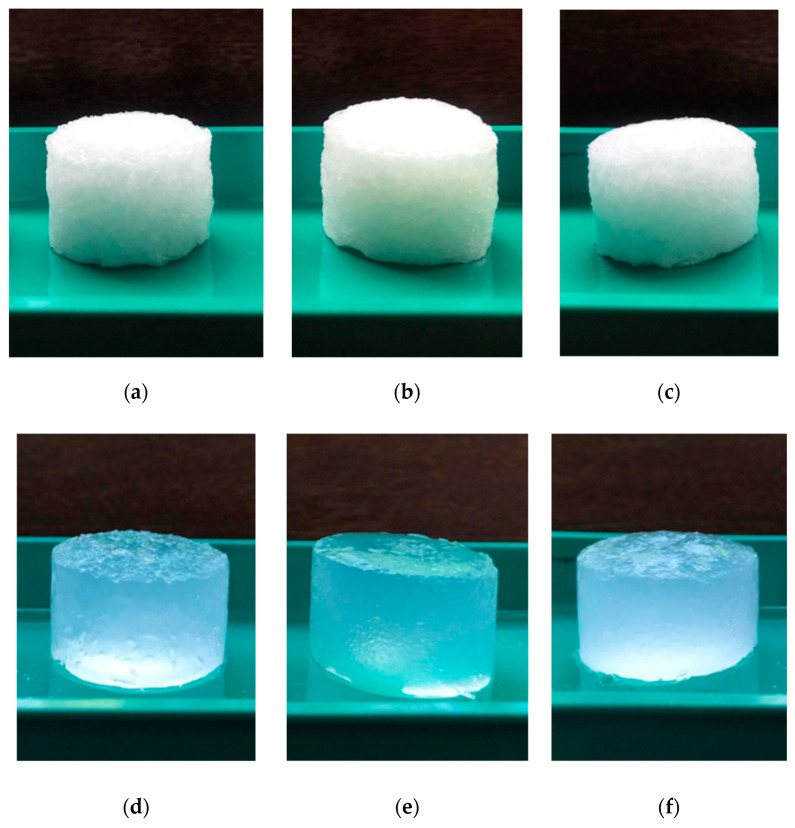
Stages in the investigation of the hydrogels based on cellulose allomorphs: (**a**–**c**) dry state of the hydrogels H-CI, H-CII, and H-CIII, and (**d**–**f**) wet state of the hydrogels H-CI, H-CII, and H-CIII.

Novel hydrogels were produced by chemical cross-linking with ECH of different cellulose allomorphs (CI, CII, and CIII), starting from a gel-like mass, obtained as an intermediary stage within the cellulose dissolution process in NaOH-water system, at low temperature (Figure 1a–c). One of the main interests of this study was to evaluate the influence of the cellulose allomorphs’ structure upon the swelling capacity of their corresponding hydrogels (Figure 1d–f) since the ability to absorb water represents one of the most important features of these 3D materials.

The three hydrogels prepared from the cellulose allomorphs H-CI, H-CII, and H-CIII showed different swelling abilities, as reflected by the different values of the maximum swelling degree (Qmax) and equilibrium swelling degree (Qeq) (Table 1).

**Figure 2:**
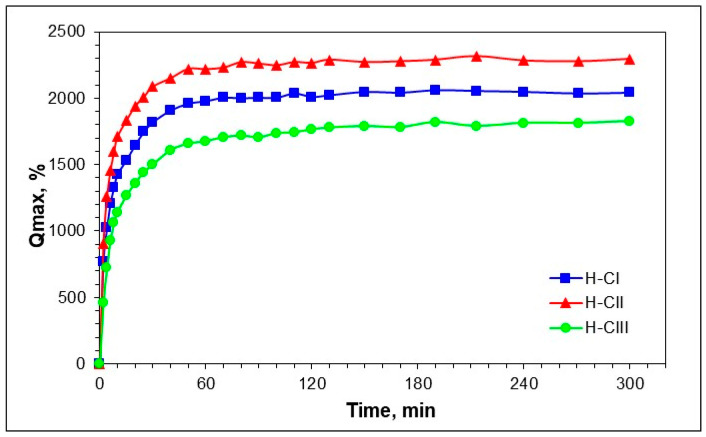
Evolution of the swelling degree (Qmax) for the hydrogels based on cellulose allomorphs H-CI, H-CII, and H-CIII.

The graph displayed in Figure 2 shows that the hydrogel H-CII, obtained from the cellulose II allomorph, exhibited the highest value of the swelling degree (Qmax = 2295%; Qeq = 2440%). A medium value was recorded for the hydrogel prepared from the CI allomorph (H-CI: Qmax = 2042%; Qeq = 2128%), while the lowest swelling values were registered for the CIII allomorph (H-CIII: Qmax = 1826%; Qeq = 1926%). The swelling occurred rapidly in the first 50 min and then slowly reached a constant value (Qmax) for all the samples. The Qeq values of the never-dried hydrogels are higher than that obtained for the Qmax of the freeze-dried hydrogels, due to the well-known hornification phenomenon [28]. However, the same descending order is maintained for both swelling degrees, such as H-CII > H-CI > H-CIII.

The dynamic swelling behavior of the hydrogels is dependent on the relative contribution of polymer relaxation, as well as of the penetrant diffusion [40,41]. In order to study the effect of the hydrogel type (H-CI, H-CII, and H-CIII) on the kinetic of water uptake process, swelling data were fitted using the power-law expression (Equation (3)) [41]. The swelling kinetic parameters of the hydrogels based on the cellulose allomorphs are presented in Table 1.

The parameter n_sw_ is the diffusion exponent that indicates the water transport mechanism, while k_sw_ is the swelling constant, which is related to the structural network. For a hydrogel, (i) n = 0.5 indicates the Fickian diffusion (case I), (ii) n = 1 implies relaxation-controlled transport mechanism (case II), and (iii) 0.5 < n < 1 indicates the non-Fickian or anomalous transport (case III) [42]. The values of n < 0.5 show a pseudo-Fickian (less-Fickian) diffusion [43]. The constants n_sw_ and k_sw_ were calculated from the slopes and intercepts of the plots of ln (Mt/M_0_) vs. ln t.

The values of n_sw_ obtained in the case of H-CI and H-CII hydrogels (n_sw_ < 0.5) indicate that the water transport mechanism follows the less Fickian diffusion, in which the rate of diffusion is much smaller than the rate of relaxation (R_diffusion_ < R_relaxation_, system controlled by diffusion) [44]. A slight increase of n_sw_ for H-CI indicated the fact that the swelling is controlled more by the polymer chain relaxation rate then the water diffusion rate. For H-CIII, it was observed that n_sw_ > 0.5 (n_sw_ = 0.614), which indicated a shift from the less-Fickian diffusion (water transport mechanism) to a non-Fickian or anomalous transport (relaxation-controlled transport mechanism). This type of diffusion indicates the fact that the water diffusion and the polymer relaxation rate are about the same order of magnitude (R_diffusion_ ~ R_relaxation_).

The swelling rate constant, k_sw_, is another important parameter that determines the diffusional characteristics of hydrogels and a slight decrease of this parameter were observed in the case of H-CI and H-CIII hydrogels.

For all the hydrogels, the correlation coefficients R^2^ are higher than 0.99, a fact that indicates the high accuracy of the linear regression equations and good agreement between the experimental data and the chosen model.

### 2.2. Morphological Investigation of the Hydrogels

SEM is a relatively simple morphological analysis technique that requires minimum sample preparation and provides solid and realistic information regarding the spatial arrangement of any given material [28]. Therefore, SEM was used to study the surface features of the hydrogels based on cellulose allomorphs and to collect data with respect to the appearance, integrity, porosity, and degree of uniformity of the hydrogels. This technique offers essential details regarding the morphological aspects of the hydrogels, which ranged from millimeters down to micrometers.

**Figure 3:**
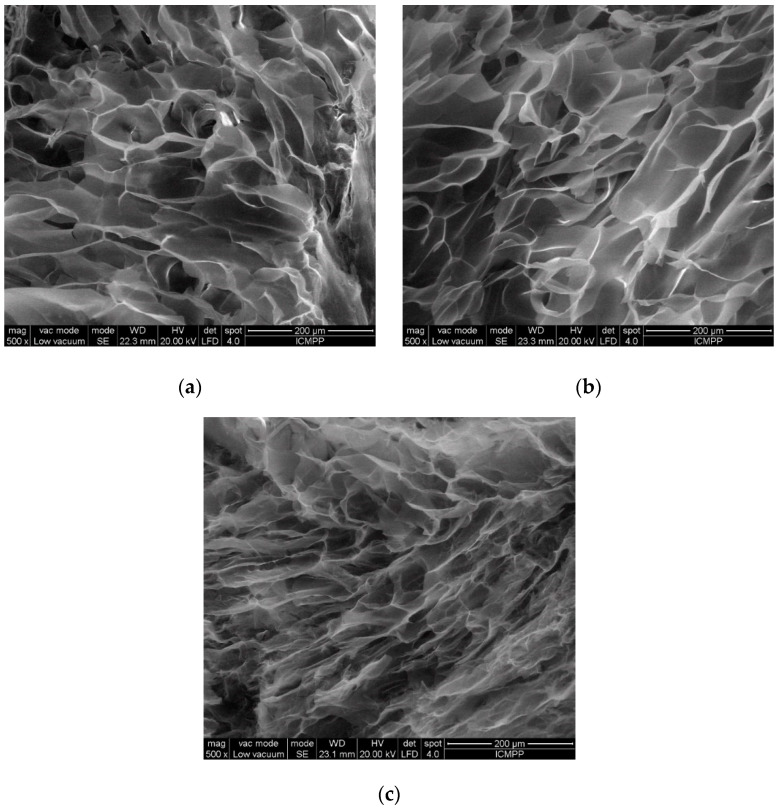
SEM micrographs of the hydrogels based on cellulose allomorphs: (**a**) H-CI, (**b**) H-CII, and (**c**) H-CIII.

It is expected that the different types of the allomorph used in the hydrogels will be reflected in different morphologies, as was also observed in the investigations on the swelling behavior (Figure 3).

The analysis of SEM micrographs evidenced the fact that the 3D polymeric matrices with dissimilar homogeneities and had varying porous structures, and they differed from one another in terms of shape, dimension, and distribution of pores. The average pore size was determined from the SEM micrographs, by measuring 50 randomly chosen pores.

The H-CI hydrogel shows interconnected pores of a roughly circular shape, dispersed in a quasi-homogeneous, compact matrix. The average diameter of the pores is 86.2 ± 12.7 μm, confirming a tightly packed morphology, a fact that is explains the relatively small amount of water absorbed by this 3D matrix, as was confirmed by the swelling data.

In comparison, the H-CII hydrogel has bigger, interconnected, and ovoid-pores distributed along a more homogeneous surface. The average diameter of these pores is 108.2 ± 15.3 μm, thus explaining the more relaxed morphology of these hydrogels, which is able to incorporate a larger quantity of water.

The H-CIII hydrogel has the most compacted pores network, with small interconnected pores (an average diameter of 67.5 ± 12.6 μm), information that correlates well with the swelling data, with H-CIII having the lowest swelling degree compared to the other two hydrogels.

All the observations from the SEM micrographs are consistent with the information data on the swelling behavior, confirming a direct dependence between the pore size and the swelling degree of the hydrogels; thus, the bigger the pore size, the higher the swelling degree.

### 2.3. Crystallinity in Cellulose Allomorphs and Cellulose-Based Hydrogels

The differences in the swelling behaviors of the hydrogels were initially attributed to the differences in the structural organization of the starting materials (cellulose allomorphs), which is why a complex investigation of the supramolecular structure by XRD of both the starting materials and the hydrogels was performed.

**Figure 4:**
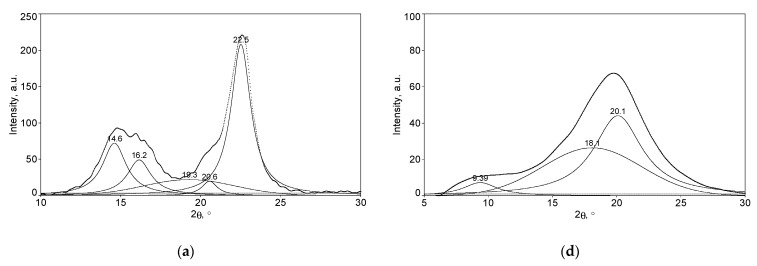

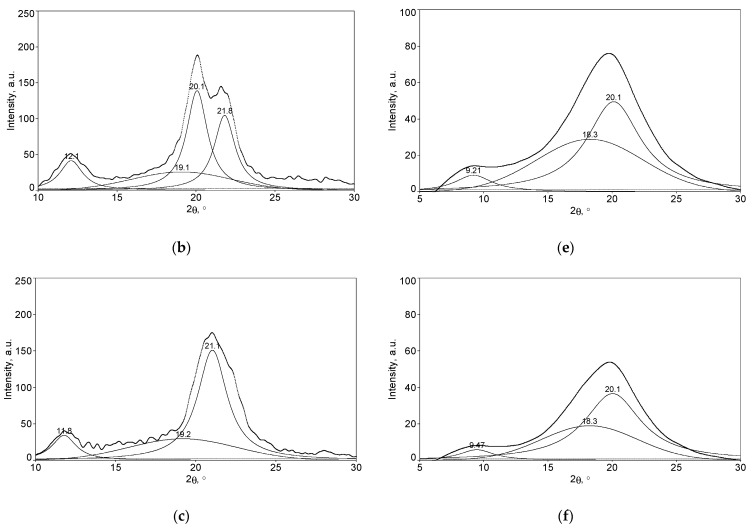
The deconvoluted XRD diffractograms of (**a**–**c**) cellulose allomorphs (CI, CII, and CIII) and (**d**–**f**) hydrogels obtained from the corresponding cellulose allomorphs (H-CI, H-CII, and H-CIII).

The XRD diffractograms characteristic of cellulose allomorphs (CI, CII, and CIII), as well as those of the corresponding hydrogels (H-CI, H-CII, and H-CIII), are presented in Figure 4. Several structural changes that occur during the mercerization process are highlighted in Figure 4 and consist of a decrease in the degree of crystallinity of CII compared to CI and a complete transformation of CI into the CII allomorph (Figure 4b).

It has been observed that the transition process from CI to CII is accompanied by modifications of the intensities corresponding to different crystallographic planes. The diffraction peaks characteristic of CI are presented in Figure 4a and appear at Bragg angles (2θ) of 14.6°, 16.2°, and 22.5°, which are typical for the crystallographic planes, (110), (1–10), and (200), respectively. In the case of the CII, three characteristic cellulose lattice planes were identified in the XRD diffraction pattern (Figure 4b), and these appear at 12.1° assigned to the (110) plane, at 20.1° for the (1–10) plane, and at 21.8° for (200) plane, respectively. Generally, CII is characterized by a slightly larger peak (1–10) on the left and a slightly smaller (200) on the right, a well-known difference that demonstrates the complete transformation of a crystalline structure of CI to a crystalline structure of CII [17]. This fact was also observed in our study, when it was found that CII contains a (1–10) peak with a much higher intensity than the (200) peak, proving a complete transformation of CI to the lower free energy crystalline pattern, CII. The diffractogram of CIII (Figure 4c) presents one peaks at 11.8° (110 plane) and an overlapping of the planes (1–10) and (200), at 2θ of 21.1°.

It is obvious that the crystalline organization of each type of allomorph has a distinctive diffraction pattern, with specific variations of both the intensities of the diffraction peaks and the positions of the Bragg angles, at which their characteristic maxima are placed.

The diffractograms displayed in Figure 4d–f show a significant decrease of the intensities of all the peaks characteristic of the main crystallographic planes, an overlapping of the planes (1–10) with (200), and a displacement of the plane (110) towards lower values of the angle 2θ. These modifications indicate that the chemical cross-linking has been achieved in all hydrogels, accompanied by the damage of the initial crystalline structure of cellulose.

The synthesis of hydrogels from different types of cellulose allomorphs proceeds with the major impairment of the crystalline organization. All the prepared hydrogels, regardless of the initial allomorph, lead essentially to the same structure specific to a predominantly amorphous cellulose, with peaks that have become wider and weaker. However, the presence of a certain crystallinity was observed, demonstrated by the presence of the peaks corresponding to the characteristic crystallographic planes of CII allomorph.

Thus, the diffraction peaks characteristic of H-CI (Figure 4d) appear at 9.39° for the crystallographic plane (110), while for the crystallographic planes (1–10) and (200) a Bragg angle of 20.1° was recorded. The same transformation was observed also for H-CII (Figure 4e), where the two characteristic peaks were identified at 2θ of 9.21° and 20.1°, while in the case of H-CIII (Figure 4f), the peaks appeared at 2θ of 9.47° and 20.1°.

XRD studies of cellulose-allomorphs-based hydrogels confirm that chemically cross-linked hydrogels undergo distinct transformations. The sharp reduction of the crystallinity recorded for the case of the chemically cross-linked hydrogels is a fact confirmed also by other authors [28]. In the initial cellulose, the chains are packed in a highly orderly manner with a compact structure, while in the case of hydrogels, the crystalline structure was destroyed by the cross-linking reaction, which disrupts the self-association and the packing of the cellulose chains, consequently leading to an increase of the sample’s hydrophilicity [20,28].

The changes recorded by the cellulose-allomorphs-based hydrogels (H-CI, H-CII, and H-CIII), regarding their crystallinity index (CrI) and the crystallite dimension (Dhkl) are presented in Table 2. The crystallinity index was determined by using Equation (4), while the crystallite size (Dhkl) in the direction perpendicular to the analyzed lattice planes with the Miller indices (hkl) was evaluated by using Scherrer’s equation (Equation (5)) [41,42].

Regarding the crystallinity index (CrI) of the allomorphic celluloses, there was a decrease in it when passing from the untreated sample of CI (CrI = 86.02%) to the mercerized CII (CrI = 82.20%) and to CIII (CrI = 70.72%). In the case of the hydrogels, the crystallinity indexes have not varied too much between the samples, with the recorded values being 44.99% for H-CI, 46.62% for H-CII, and 40.71% for H-CIII.

A relationship between the crystallinity index and the crystallite dimensions was observed for the studied samples. Thus, the smaller the dimensions of the crystallites, the lower the CrI of the sample, a fact associated with an increase of the amorphous domains. This aspect was also confirmed by other authors [45,46].

Moreover, the dramatic decrease in the crystalline indexes of cellulose-based hydrogels is a result of chemical crosslinking between the hydroxyl groups of the cellulose chains.

However, XRD investigations have shown the same type of supramolecular organization of hydrogels, regardless of the type of allomorph used as starting material, which does not explain the differences in the swelling behavior of hydrogels resulting from different allomorphs.

### 2.4. Rheological Evaluation of the Gels and Hydrogels Based on Cellulose Allomorphs

Since the changes in the supramolecular structure do not explain the swelling behavior, the explanation for the differences in the swelling behavior was searched for in the differences recorded during the hydrogel synthesis processes.

Hydrogels were prepared starting from a swelling stage of cellulose in NaOH solutions, at low temperatures, based on a method developed by Isogai and Atalla [39], followed by chemical cross-linking with epichlorohydrin (ECH).

**Figure 5:**
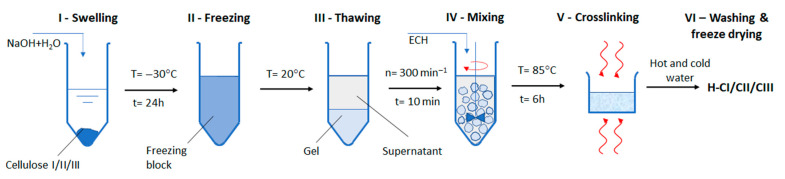
Schematic method for the preparation of hydrogels based on cellulose allomorphs.

As already known, the first stage in polymer dissolution is swelling (Figure 5—stage I), a phenomenon based on the incorporation into the polymer of a large amount of solvent. The swelling may be limited or unlimited, similar to dissolution. The limited swelling is the process of interaction of the polymer with the solvent, which is stopped when the latter is absorbed by the polymer, without a spontaneous dissolution. The method of dissolving cellulose at low temperatures involves a complete freezing stage by cooling to very low temperatures (Figure 5—stage II), and the thawing of the obtained block allows one to obtain the gel phase. Two phases are specific to the limited swelling process: one represented by the polymeric matrix in which the solvent molecules diffused—*the gel*—and other represented by the pure solvent or diluted solution of polymer in solvent—*the supernatant*. The two phases are delimited by a separation surface and they coexist in an equilibrium, as shown in Figure 5—stage III.

**Figure 6:**
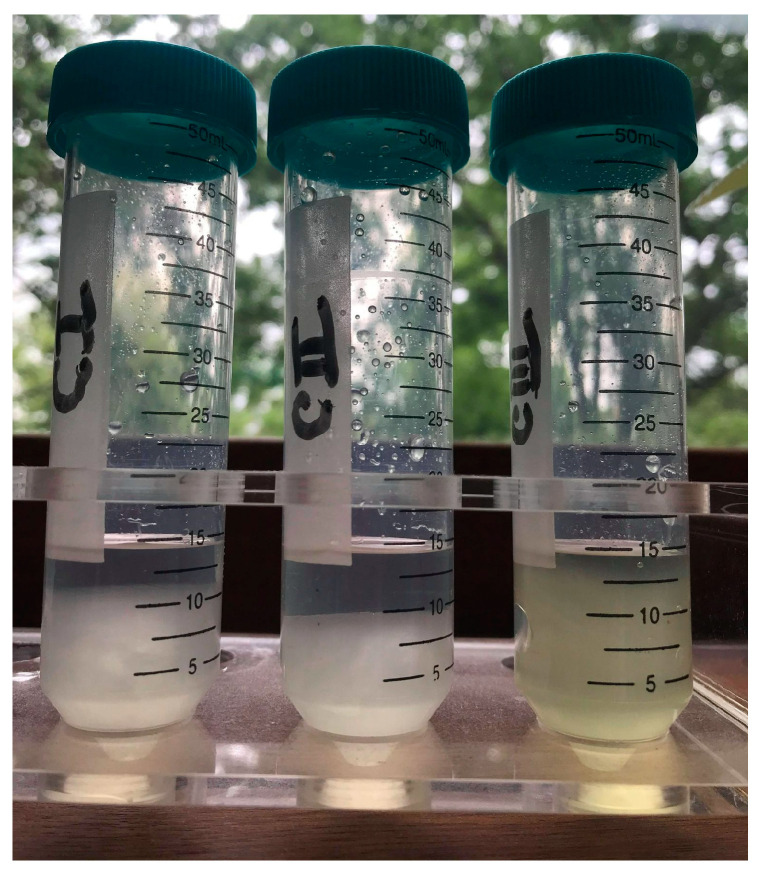
Gel phases corresponding to each type of cellulose allomorphs: CI, CII, and CIII.

The first differences appeared in the aspect of the gel phase resulting from the swelling in NaOH solution of cellulose allomorphs (Figure 6).

The gels have different appearances, volumes, consistencies, and densities. The density and polymerization degree corresponding to the cellulose allomorphs are presented in Table 3.

The gel resulting from the swelling of the CII allomorph is obviously the most compact, while that of the CIII allomorph is the least dense. These aspects were reflected in their behavior in the next stage, the mixture with the cross-linker under controlled shear regimes.

**Figure 7:**
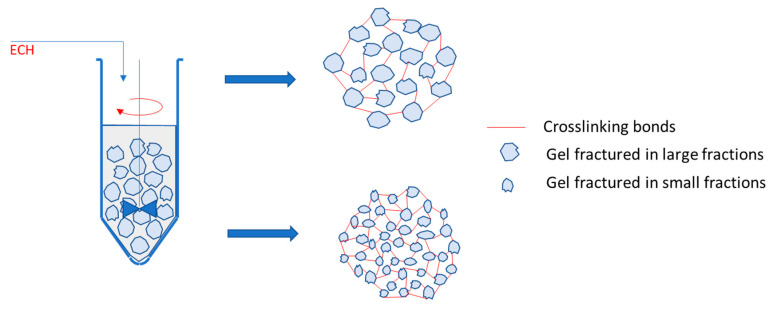
A model for the hydrogels synthesis, as a result of the cross-linking of gels fragmented in large or small fractions.

By mixing with the cross-linking agent in identical shear regimes, the cellulose-NaOH gels are fractured into fragments of sizes correlated with the strength of the gels. The result is a hydrogel with a high cross-linking density if the gel fragments are small in size and with a lower cross-linking density in the case of resistant gels that fracture into large fragments (Figure 7).

In order to validate the proposed model, rheological investigations were performed, which were able to explain both the structure and the swelling behavior of the obtained hydrogels.

To highlight the strength of cellulose-NaOH gels, oscillatory amplitude sweeps tests were carried out. The linear viscoelastic region (LVR) was determined, and the brittle fracturing behavior of the gels was evidenced.

The (elastic) storage modulus G′ represents the elastic portion of the viscoelastic behavior, while the (viscous) loss modulus G″ characterizes the viscous portion of the viscoelastic behavior.

The approximate values of G′ within the LVR, which refer to the gel strength, vary between 6350 Pa for CI, 11,500 Pa for CII, and 3500 Pa for CIII, showing enhanced stiffness “at rest” of the network (G′LVR) for cellulose allomorphs CII followed by CI.

When the LVR is exceeded, G′ and G″ are dependent on the strain amplitude, usually decreasing with its enhancement for all gels at large deformations. The amplitude sweep results reveal the network breakdown as the strain increases. All the studied gels of cellulose allomorphs exhibit a “flow point” (crossover G′ = G″) over 14.5%. The stress and strain at the flow points (δ_f_) and (γ_f_), respectively, were determined at the crossover point (G′ = G″), with the resulting values being presented in Table 4 [47].

The experimental results show a lower strength of CIII at the flow point, with the lowest values of the dynamic moduli, δ_f_ and γ_f_, and the maximum structure breakdown of this cellulose allomorph being registered at 14.54% strain deformation.

**Figure 8:**
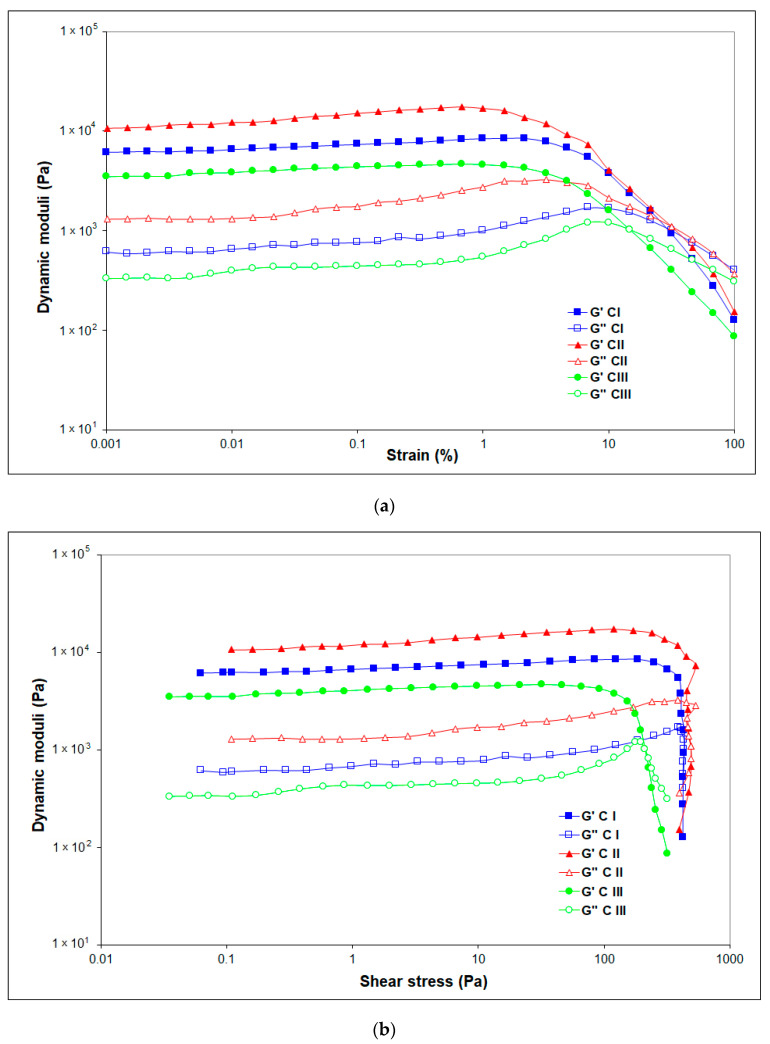
(**a**) Amplitude sweep test results (**a**) G′ and G″ dependence of the oscillatory strain for CI, CII and CIII; and (**b**) G′ and G″ dependence of the shear stress for gels CI, CII, and CIII.

Figure 8 shows that G′ displays the sharpest downturn starting at γ = 1% for CII, thus indicating a brittle fracturing behavior that led to the inhomogeneous breaking of gel CII under shear into larger pieces [48].

Moreover, Figure 8 displays that the loss modulus G″ follows an almost constant value in the LVE region for all gels, but, unlike G′, the G″ curve rises sharply because initially a few individual bonds in the network are broken, developing micro cracks, keeping the entire surrounding material firmly together. After reaching the maximum peak before breakdown, the gel starts to flow so the G″ curve drops continuously and not as steep as G′. The sharp increase of the viscous modulus G″ is due to the internal viscous friction of the resulting freely movable fragments around the micro cracks (Figure 8b). The individual micro cracks continue to grow and might lead to the formation of the continuous macro crack through the entire material that starts to flow, with the viscous behavior of the sample dominating in this case (G″ > G′) [49].

The shape of G″ can be used to distinguish the behavior of the gels. The process of structural breakdown is faster for CII and delayed for CI (and especially CIII).

**Figure 9:**
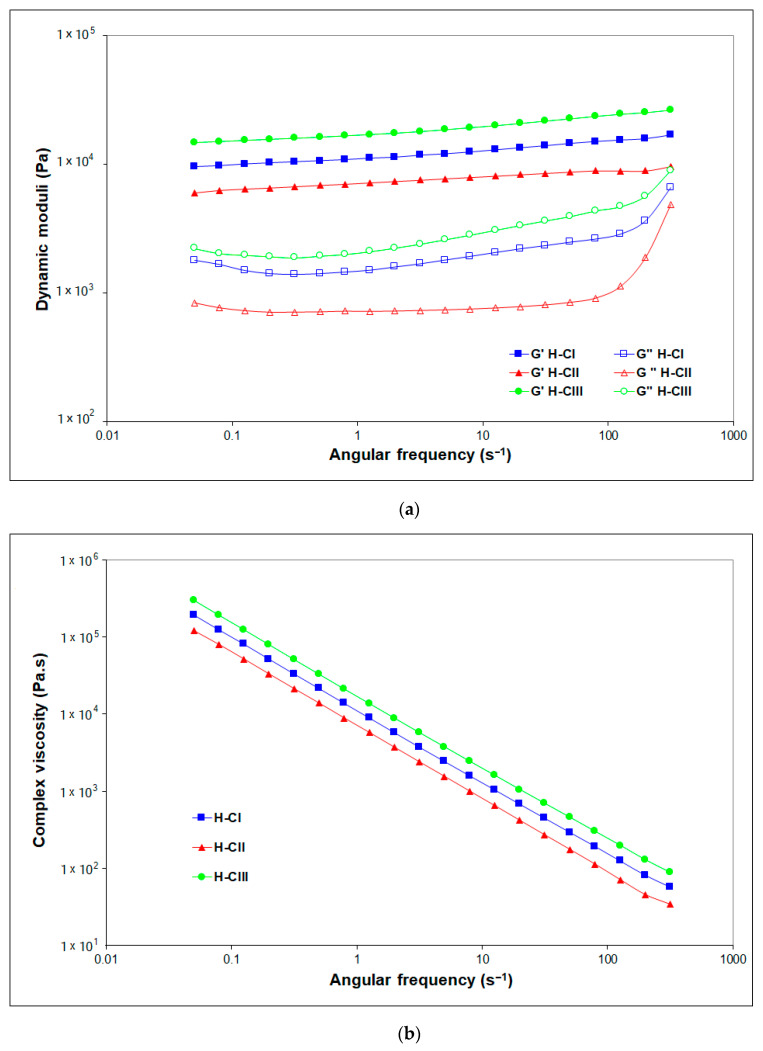
Frequency sweep tests results of hydrogels: (**a**) the dependence of the storage modulus G′ (solid symbols) and loss modulus G″ (open symbols) on the angular frequency; (**b**) the dependence of the complex viscosity on the angular frequency.

The rheological measurement results offer information on the structure–property relationship of the hydrogels. The dynamic viscoelastic properties of the formed networks of cellulose-based hydrogels were evaluated by frequency sweep analysis (Figure 9). The elastic features of the matrix were dominant throughout the whole measured angular frequencies, characterized by G′ values higher than G″ values, which demonstrates typical gel-like behavior.

All values, especially for G′, are almost independent of the tested angular frequency, proving that the hydrogels display an excellent structured three-dimensional (3D) network, and the applied deformation does not affect the stability of the studied hydrogels’ network structure. The absence of a crossover frequency indicates permanent chemical cross-linking.

The graph shown in Figure 9a reveals that the structure of H-CII is softer, with a lower resistance of the hydrogel (G′) over the tested oscillation frequencies compared with H-CIII, but its stable structure is maintained. At higher angular frequencies (over 100 s^−1^), the loss (viscous) moduli increase, although not overlapping the storage moduli, maintains the gel-like behavior up to 500 s^−1^, the highest tested angular frequency.

The hydrogel corresponding to cellulose allomorph III (H-CIII) exhibits a higher storage and loss modulus than hydrogels H-CI and H-CII, showing a stronger and stiffer structure. This mechanical feature is translated also into a higher curve of complex viscosity for H-CIII (Figure 9b). The complex dynamic viscosity decreases with the increasing oscillation frequency for all the evaluated cellulose-based hydrogels.

All the rheological results are in good correlation with the SEM and XRD data and with those obtained for the swelling ability of the hydrogels.

### 2.5. ATR-FTIR Analysis of the Cellulose Allomorphs and Cellulose-Based Hydrogels

ATR-FTIR spectroscopy was used in order to prove the achievement of the cross-linking reaction between cellulose and EPC, this being a highly effective analytical tool for the evaluation of the surface chemical composition of materials.

**Figure 10:**
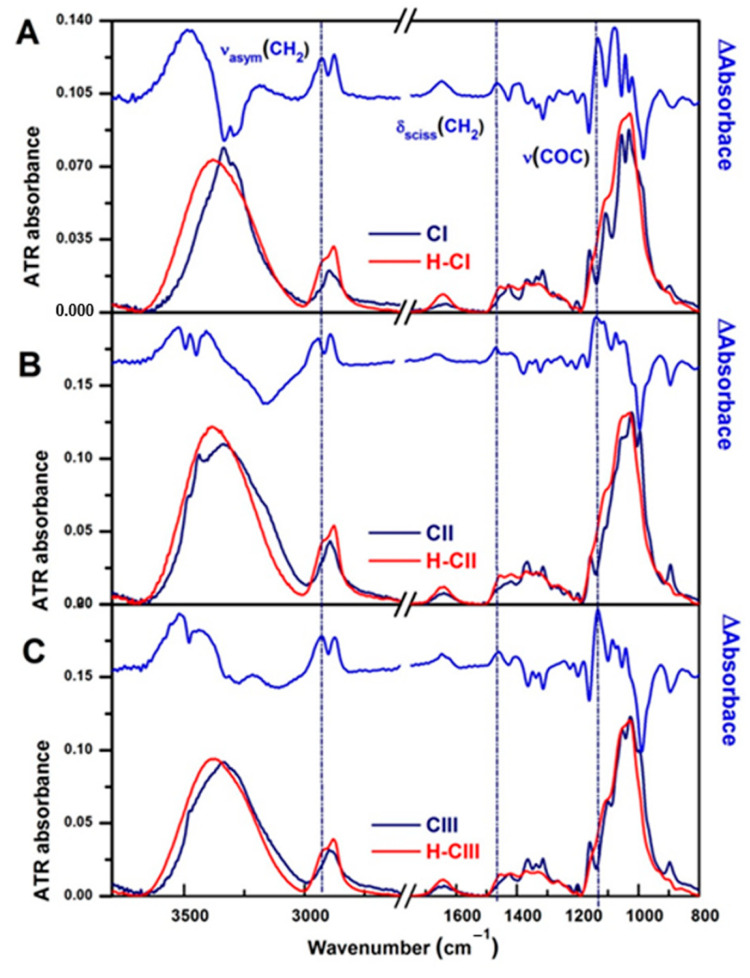
ATR-FTIR spectra comparison of cellulose allomorphs (dark blue line) with those of their corresponding hydrogels (red line): (**A**) CI and H-CI, (**B**) CII and H-CII, and (**C**) CIII and H-CIII. The difference spectrum [H-Ci – Ci] is added to the top of each figure (blue line). The vertical dotted lines mark the position of the characteristic bands of the chemical cross-linking of cellulose with ECH.

The ATR-FTIR spectra of the hydrogels (H-CI, H-CII, and H-CIII) compared with their corresponding cellulose allomorphs (CI, CII, and CIII) are shown in Figure 10.

The spectral modification in the region 1500–800 cm^−1^ is assigned to an alteration of the crystalline structure of cellulose, following the alkaline treatment and, implicitly, the chemical cross-linking of cellulose with ECH [50]. In addition, it can be observed that the band at 1636 cm^−1^, characteristic of the adsorbed water [51], is more intense in the hydrogel spectra. This observation reflects the higher hydrophilic character of the hydrogels in comparison with their corresponding cellulose allomorphs.

The difference spectrum [H-C_i_ – C_i_] has been calculated in order to highlight the spectral changes resulting from chemical cross-linking (top spectra in Figure 10).

All the three allomorphic forms of cellulose present the decrease of the bands at:

− 994–980 cm^−1^ (ν(CO) at C6) with the counterpart at 3437 cm^−1^ (O2-H⋯O6 intramolecular H-bonds);
− 1054 cm^−1^ (ν(CO) at C3), 3339 cm^−1^ (O3-H⋯O5 intramolecular H-bonds), and 3301 cm^−1^ (O6-H⋯O3′ intermolecular H-bonds);
− 1106 cm^−1^ (ν(CO) at C2) and 3296 cm^−1^ (coupled vibrations of O2-H⋯O6 and O3-H⋯O5 intramolecular H-bonds) [51,52,53,54].

These changes show that in all three hydrogels, the chemical crosslinking of cellulose with ECH takes place mainly at the secondary alcohol C2 and the primary alcohol C6 and less at the secondary alcohol C3. The same observations were also confirmed by other authors [38,55].

This finding can be explained by the fact that Na^+^ breaks the intermolecular hydrogen bonds O2-H⋯O6′ and that the most probable location of the Na^+^ ion is at C2, thus facilitating the realization of the cross-linking reaction on this site [56,57,58,59]. In addition, many studies show that C3 carbon is the most resistant to complexation with NaOH [30], which is also observed in our case.

In the case of CI, the large band at 1102 cm^−1^ indicates that the secondary alcohols C2 contributed to the formation of ether-based cross-links. Besides the very clear ν(O2-H⋯O6) peak at 3437 cm^−1^, the initial CII cellulose had an additional peak at 3480 cm^−1^, which belongs to ν(O6-H) weakly H-bonded and to some –OH groups located in the interface and surface regions of crystallites [60]. The disappearance of both peaks upon crosslinking certifies the formation of ether-based linkage in the less organized regions first and also attests to the disruption of intermolecular hydrogen bonds (O6-H⋯O3′). In the case of H-CIII, the intense negative band at 1160 cm^−1^, characteristic of ν(COC) of glycosidic linkage, shows that the network formed through cross-linking reaction has decreased its degrees of freedom.

In the spectra of the three hydrogels, H-CI, H-CII, and H-CIII, the chemical crosslinking of cellulose with ECH is highlighted by the formation of new bands at 2930 and 2873 cm^−1,^ assigned to ν_asym_(CH_2_) and ν_sym_(CH_2_); at 1458 cm^−1,^ given by the δ_sciss_(CH_2_), and at 1133 cm^−1,^ assigned to ν(COC) of the ether linkages [61].

Because the FTIR spectra of the three hydrogels are very similar and the ν(OH) vibration has the same position and profile in the all three spectra (~3380 cm^−1^), the network ultimately has the same structure irrespective of the type and number of alcohol groups involved.

## 3. Conclusions

Cellulose-based hydrogels were prepared from three allomorphic forms of cellulose (CI, CII and CIII) starting from a gel stage, within their dissolution process in NaOH-water system, followed by the chemical cross-linking with ECH. Several correlations have been established by means of the swelling behavior, XRD, SEM, ATR-FTIR, and rheological measurements.

SEM investigations evidenced 3D networks with dissimilar morphologies and homogeneities, depending on each cellulose allomorph, as well as a close correlation between the type of cellulosic allomorph, swelling degree, and morphology of the porous structure.

The method of hydrogel synthesis proceeds with a major impairment of the crystalline organization, even if the dissolution process is partial, being limited only to the swelling phase. All hydrogels prepared regardless of the starting allomorph led to essentially the same supramolecular organization, specific to mostly amorphous cellulose.

The gel stage in the synthesis of hydrogels from cellulose allomorphs plays a key role in obtaining hydrogels with different performances. By swelling in NaOH solutions, at a low temperature, cellulose allomorphs led to gels with different strengths and rheological characteristics. By mixing with the cross-linking agent in identical shear regimes, the cellulose-NaOH gels were fractured into fragments of sizes correlated with the strength of the gels. The result was a hydrogel with a high cross-linking density, if the gel fragments were small in size and with a lower cross-linking density, in the case of resistant gels that fractured into large fragments.

It has been shown that in order to prepare hydrogels with higher characteristics than those obtained from CI, by the method used in this study, a previous conversion of CI to the CII allomorph is necessary. By swelling in NaOH-water, at low temperatures, CII leads to denser gels, which are more difficult to fragment and, implicitly, to less cross-linked hydrogels (H-CII), which have superior swelling capacity. In the case of the CIII allomorph, the obtained hydrogels (H-CIII) demonstrated inferior swelling characteristics to those of native cellulose (H-CI) but superior rheological and resistance properties.

In conclusion, some of the most important features of these hydrogels (controlled porosity and swelling degree) can be tailored by using a certain type of cellulose allomorph (C I, CII or CIII), in order to fulfill a certain set of characteristics requested by a given application (wound dressing or tissue engineering).

## 4. Materials and Methods

### 4.1. Materials

Cellulose I (CI), the microcrystalline cellulose, was purchased by Sigma-Aldrich (Saint Louis, MO, US) under the trade name of Avicel PH-101. The cross-linking agent, epichlorohydrin (ECH), was purchased from Merck (Hohenbrunn, Germany) and was used without further purification. Sodium hydroxide (NaOH) in pellets, with a purity ≥ 97%, was supplied by Merck (Hohenbrunn, Germany).

### 4.2. Preparation of Cellulose Allomorphs

Cellulose II (CII), mercerized cellulose, was prepared from cellulose I by soaking in a 17.5% NaOH solution for 24 h at 20 °C, followed by a rigorous rinsing with distilled water and drying in vacuum. Cellulose III (CIII) was prepared by soaking cellulose I in organic amine (100% ethylenediamine) for 24 h, at room temperature. The cellulose amine complex was washed with anhydrous methanol, and finally cellulose III samples were dried in vacuum.

### 4.3. Preparation of Cellulose Hydrogels

Hydrogels based on cellulose allomorphs were prepared by a patented procedure [62]. One g of cellulose was suspended in 26.9 mL of water, 2.5 g of NaOH was then added, and the mixture was shaken to dissolve the NaOH at room temperature, resulting in a suspension of the cellulose in an 8.5% NaOH solution. The suspension was cooled to −30 °C, up to a frozen solid, and held at that temperature 24 h. The frozen solid was then allowed to thaw at room temperature and was transformed into a gel-like mass, and the epichlorohydrin was added under controlled stirring, for 10 min. The obtained composition was cross-linked for 6 h at 85 °C. The obtained hydrogels were washed several times with warm water in order to remove the excess of salts and any traces of the cross-linking agent from their structures. The obtained hydrogels were dried in a freeze-dryer and were coded like H-CI for the one obtained from cellulose I and, respectively, H-CII and H-CIII for those obtained from cellulose II and cellulose III.

### 4.4. Degrees of Polymerization

Degrees of polymerization of cellulose (DP) were measured by the viscosity method in 0.5 mol Cuen [63].

### 4.5. Swelling Behavior

The swelling studies of the hydrogels were performed by direct immersion in distilled water, at 37 °C. The hydrogels were periodically removed from the solution, gently wiped with a soft tissue in order to remove the excess surface solution, weighed, and then placed back into the vessel. The swelling degree (Q_max_, %) of the hydrogels in distilled water was determined gravimetrically, using the following relation:

(1) $$Q_{\max}\;\left(\%\right)=\frac{m_{t}-m_{0}}{m_{0}}\times 100$$

where m_t_—the weight of the swelled hydrogel at time t; and m_0_—the weight of dry hydrogel.

The equilibrium swelling degree (Q_eq_, %) was determined for the never-dried hydrogels and was calculated by using the following equation:

(2) $$Q_{\mathrm{eq}}\;\left(\%\right)=\frac{m_{\infty }-m_{0}}{m_{0}}\times 100$$

where: m_∞_—the weight of the swelled hydrogel at equilibrium; and m_0_—the weight of dry hydrogel.

To determine the kinetics of solvent diffusion into the matrices (swelling), the following equation was used [41]:

(3) $$\frac{W_{t}}{W_{\mathrm{eq}}}=k_{\mathrm{sw}\;}\times {\;t}^{n_{\mathrm{sw}}}$$

where W_t_—the amount of water absorbed by the hydrogel at time t; W_eq_—the amount of water absorbed by the hydrogel at equilibrium; k_sw_—the swelling constant incorporating characteristics of the macromolecular network system (min^−n^); and n_sw_—the swelling diffusional exponent, which is indicative of the transport mechanism.

The constants n and k were calculated from the slopes and intercepts of the plots of ln (W_t_/W_eq_) vs. lnt. Equation (3) was applied in the early swelling stages (swelling degree less than 60%), and the linearity was observed.

### 4.6. X-ray Diffraction Analysis (XRD)

XRD investigations were performed by using a Bruker-AXS D8 ADVANCE (Bruker AXS GmbH, Karlsruhe, Germany) apparatus, having a transmission type goniometer based on Ni-filtered, Cu-Kα radiation at 40 kV. The goniometer was scanned stepwise every 10° from 10° to 40° within the 2θ range. The resulting diffraction patterns exhibited peaks that were deconvoluted from a background scattering by using Lorenzian functions, while the diffraction pattern of an artificially amorphized sample was approximated by a Gaussian functions curve fitting analysis [17,23]. The data were obtained using XRD Commander software (Bruker AXS GmbH, Karlsruhe, Germany) and were processed with DIFFRAC.EVA V1.1 (Bruker AXS GmbH, Karlsruhe, Germany). The crystallinity index (CrI) was calculated by using the following equation [23]:

(4) $$\mathrm{CrI}\;\left(\%\right)=\frac{S_{C}}{S_{C}+{\;S}_{A}}\times 100$$

where S_C_ and S_A_ are the surfaces of the crystalline and amorphous areas, respectively.

The crystallite sizes were measured from their respective XRD patterns by using the Scherrer equation [23]:

(5) $$D_{\mathrm{hkl}}=\frac{k\times \lambda }{\beta \times \cos\theta }$$

where D_hkl_ is the size of crystallite (nm), k is the Scherrer constant (0.94), λ is the X-ray wavelength (0.15418 nm), β is the full-width at half-maximum of the reflection *hkl*, and θ is the half of the 2θ value for the corresponding Bragg peak.

### 4.7. Scanning Electron Microscopy (SEM)

A thorough analysis of the internal morphology of the hydrogels was performed by using a scanning electron microscope Quanta 200 (FEI Company, Hillsboro, OR, USA) operating at 20 kV with secondary electrons, in low vacuum mode. SEM investigations were focused on the morphological properties of the hydrogels, including homogeneity, sample shape, surface changes, and porosity. The hydrogels samples were studied at various magnifications, with each magnification being mentioned additionally to the corresponding SEM micrograph.

### 4.8. Rheological Characterization

A stress-controlled Rheometer Physica MCR-301 (Anton Paar, Graz, Austria) has been used to investigate the rheological properties of cellulose allomorphs gels and their corresponding hydrogels. Specific oscillatory measurements were performed in dynamic mode using parallel plate geometry with 25 mm diameter and a 3 mm gap between the plates. Each swollen sample was placed on the lower plate, the upper plate was lowered to the desired gap height and the sample diameter was cut to fit the used plate geometry, removing the excess sample. In order to prevent the water evaporation, the rheological measurements were conducted at a constant temperature (25 ± 0.1 °C), and a special system for controlling temperature and evaporation was used. Oscillatory amplitude sweeps tests (strain between 0.001% and 100% at 10 rad/s) were carried out to determine the linear viscoelastic region (LVR) and to characterize the gels’ microstructure and the fracture process of the gels. The viscoelastic properties of the cellulose-based hydrogels were investigated by frequency sweep tests in the frequency range from 0.05 to 500 s^−1^, with a constant strain of 1%.

### 4.9. Attenuated Total Reflectance Fourier Transform Infrared (ATR-FTIR) Spectroscopy

A Vertex 70 spectrometer (Bruker, Hamburg, Germany) was used for the Fourier transform infrared (FTIR) spectra recording in the attenuated total reflection (ATR) configuration. All spectra were collected at 128 scans, at a 2 cm^−1^ resolution, in the mid IR range (4000–600 cm^−1^). The OPUS 6.5 software was used for the FTIR data processing.

## Figures and Tables

**Table 1:** Swelling kinetic parameters of cellulose-allomorphs-based hydrogels.

Sample	Swelling Kinetic Parameters			Qmax (%)	Qeq (%)
	k_sw_	n_sw_	R^2^		
H-CI	0.291	0.386	0.999	2042	2128
H-CII	0.321	0.377	0.999	2295	2440
H-CIII	0.165	0.614	0.999	1826	1926

**Table 2:** Crystallite size (D) and crystallinity index (CrI) of the cellulose allomorphs and cellulose-based hydrogels.

Samples		D_(110)_ (nm)	D_(1–10)_ (nm)	D_(200)_ (nm)	CrI (%)
Cellulose allomorphs	CI	5.995	5.232	8.330	86.02
	CII	5.492	7.786	8.191	82.20
	CIII	4.821	8.039	8.039	70.72
Cellulose-based hydrogels	H-CI	3.615	2.665		44.99
	H-CII	4.349	3.147		46.62
	H-CIII	3.009	2.636		40.71

**Table 3:** The density and polymerization degree corresponding to the cellulose allomorphs.

Sample	CI	Alkali-Treated CI	CII	Alkali-Treated CII	CIII	Alkali-Treated CIII
DP	183	171	154	149	164	158
Density (g/mL)	1.050	-	1.282	-	0.960	-

**Table 4:** The values of dynamic moduli at the crossover point (G′ = G″), and the stress and strain at the flow point (δ_f_ and γ_f_).

Sample	G′ = G″ (Pa)	δ_f_ (Pa)	γ_f_ (%)
CI	1072	434.9	28.57
CII	1102	491.1	31.52
CIII	1027	211.4	14.54

## Data Availability

Not applicable.
